# Anti-hyperglycaemic and lipid lowering potential of *Adenanthera pavonina* Linn. in streptozotocin induced diabetic rats

**DOI:** 10.1007/s13596-012-0074-2

**Published:** 2012-05-24

**Authors:** Ramdas B. Pandhare, B. Sangameswaran, Popat B. Mohite, Shantaram G. Khanage

**Affiliations:** 1 MES College of Pharmacy, Sonai, Newasa, Ahmednagar, Maharashtra 414105 India; 2 Adesh Institute of Pharmacy and Biomedical Sciences, Bathinda, Punjab India; 3 Department of Pharmacy, Suresh Gyan Vihar University, Jagatpura, Jaipur India

**Keywords:** Adenanthera pavonina, Streptozotocin, antihyperglycaemic, Lipid lowering, HbA1c

## Abstract

In India, Adenanthera pavonina is traditionally used in the treatment of diabetes mellitus and lipid disorders. In the present study, the antihyperglycaemic and lipid lowering effect of Adenanthera pavonina seed aqueous extract (APSAE) was evaluated using streptozotocin induced diabetes in rats. Streptozotocin was given at the dose of 55 mg/kg, i.p. After induction of diabetes, APSAE was administered for 30 days p. o. and simultaneously different biochemical parameters like plasma glucose, HbA1c, serum triglyceride, cholesterol, LDL-cholesterol and HDL-cholesterol were estimated. Diabetic control showed significant increase (*P* < 0.01) in plasma glucose, serum triglyceride, cholesterol, LDL-cholesterol and significant decrease (*P* < 0.01) in serum HDL-cholesterol and HbA1c. Treatment with APSAE showed significant reduction (*P* < 0.01) in plasma glucose when compared with diabetic control. The elevated levels of serum triglyceride and cholesterol levels were significantly reduced (*P* < 0.01) by APSAE. APSAE treatment for 30 days showed significant decrease in serum LDL-cholesterol (*P* < 0.01) and significant increase in serum HDL cholesterol level (*P* < 0.01). Moreover, diabetic control there was significant decrease in HbA1c which was significantly increased (*P* < 0.05) by treatment with APSAE. Hence, from the result obtained in the present study it can be confirmed that Adenanthera pavonina has the potential to treat diabetes condition and associated lipid disorders.

## Introduction

Diabetes mellitus (DM) is a chronic disease caused by inherited or acquired deficiency in insulin secretion and by decreased responsiveness of the organs to secreted insulin (Matsui et al. 2007). DM is currently one of the most costly and burdensome chronic diseases and is a condition that is increasing in epidemic proportions throughout the world (King et al. 1998). Diabetes affects about 5 % of the global population (WHO traditional medicine strategy 2005) and the management of diabetes without any side effects is still a challenge to the medical system (Chakraborty and Rajagopalan 2002; Kameswararao et al. 2003). Renewed attention in recent decades to alternative medicines and natural therapies has stimulated a new wave of research interest in traditional practices. The plant kingdom has become a target for the search for new drugs and biologically active “lead” compounds (Evans 1996). Ethno botanical information indicates that more than 800 plants are used as traditional remedies for the treatment of diabetes (Pushparaj et al. 2000; Alarcon-Aguilara et al. 1998), but only a few have received scientific scrutiny.

*Adenanthera pavonina* Linn. (Family: Leguminosae-Mimosaceae), is a deciduous tree, 18–24 m tall, bole erect and 60 cm in diameter (Bouquet and Debray 1974). Many species of adenanthera, including *Adenanthera pavonina*, have been used as traditional herbal medicine against a variety of diseases including diabetes, lipid disorders, diarrhoea, haemorrhage from the stomach, haematuria and as anti-inflammatory agent in gout. The seed contains an anti-inflammatory active principle, O-acetylethanolamine. The leaves contain octacosanol, dulcitol, glucosides of betasitosterol and stigmasterol. The bark contains sitgmasterol glucoside (Khare 2007). Traditionally, the ground seed is widely used for the treatment of various human ailments such as treatment of boils, inflammation, blood disorders, arthritis, rheumatism, cholera, paralysis, epilepsy, convulsion, spasm and indigestion (Burkill 1966; Balogun and Fetuga 2004). Phytochemically, the seed and pod contain glycosides, saponins and steroids (Howes 1974; Yadav et al. 1976). A new five-membered lactone ring compound, pavonin was isolated from the methanol soluble part of *A. pavonina* (S.A.Muhammad et al. 2005), oil extracted from the seed has been reported to have membrane-stabilizing activity by reducing lytic effect on erythrocytes, exhibited by many intravenous drugs (Anna et al. 2006). The methanol seed extract has also been reported to demonstrate anti-inflammatory and analgesic activities (Olajide et al. 2004).

On the basis of reported activities and chemical constituents the aqueous extract of seed was chosen. Therefore, taking into consideration the reported pharmacological activities of *Adenanthera pavonina* Linn. the present study is planned to investigate antihyperglycaemic and lipid lowering potential in Stz- induced diabetic rats.

## Materials and methods

### Plant material

Seeds of *Adenanthera pavonina* were collected during March 2009 from the Mahatma Phule Krishi Vidyapeeth, Rahuri, Maharashtra, India. The plant was authenticated by Dr. P.G. Diwakar, Joint Director, Botanical Survey of India, Pune as *Adenanthera pavonina* Linn. (Mimosaceae) with a voucher specimen (BSI/WRC/Tech/2010/463) kept in herbarium, BSI, Pune.

### Preparation of extract

The seeds were washed with distilled water, shed dried and latter powdered. This powder was then defatted with petroleum ether which was further macerated with distilled water for 72 h with occasional shaking. It was then filtered and evaporated. The yield of APSAE was 2.5 % w/w.

### Preliminary phytochemical screening

The preliminary phytochemical screening of APSAE was carried out for qualitative identification of type of phytoconstituents present (Latha and Pari 2004; Kokate 1994).

### Animals

Healthy adult male wistar rats weighing 150-200 g and Swiss albino mice weighing 25–30 g were obtained from in house breed at the animal house of M.E.S. College of Pharmacy, Sonai and were housed in polypropylene cages lined with husk in standard environmental conditions (Temperature 25 ± 2°C; relative humidity 55 ± 10 %; and 12:12 light: dark cycle,). The animals were fed on a standard pellet diet (Amrut rat and mice feed, Sangli, India) and water ad libitum*.*

Animals were acclimatized to the laboratory condition for at least 8 days prior to the experiment and were maintained in a well ventilated animal house. The experimental protocol was approved by the Institutional animal Ethical Committee (MESCOP/IAEC/07/2010) and the care of the laboratory animals was taken as per the current CPCSEA regulation.

### Experimental design

#### Acute toxicity study (OECD 425, 2001)

Acute toxicity of APSAE was done using Swiss albino mice (25–30 g) according to the procedure of Organization for Economic Co-operation and Development (OECD) guideline no. 425 (OECD, 2001). The animals were fasted overnight prior to the experiment and maintained under standard conditions. Animals were observed for general behavioral change and mortality for a period of 14 days post treatment.

#### Effect of APSAE on normoglycaemic rats

The rats were divided into four groups of 6 animals (*n* = 6) each. Group I served as control and received distilled water. Group II, III and IV received APSAE orally at doses 50,100 and 200 mg/kg/day b.wt. Blood glucose levels were determined at 0,1,2,3 and 4 h following treatment by retro-orbital plexus of the eye under mild ether anesthesia.

#### Oral glucose tolerance test in normal rats (OGTT)

The rats were divided into four groups of 6 animals (*n* = 6) each. Group I served as control and received distilled water. Group II, III and IV received APSAE orally at doses 50,100 and 200 mg/kg/day b.wt. All the animals were given glucose (2 g/kg) 30 min after dosing. Blood samples were collected from the retro-orbital plexus of the eye just prior (0 h) and 1, 2, 3 and 4 h. after the glucose loading and blood glucose levels were estimated.

#### Induction of diabetes

Diabetes was induced in rats by single intraperitoneal injection of STZ (55 mg/kg b.wt.) dissolved in freshly prepared 0.01 M citrate buffer, pH 4.5. (Gupta et al. 2004) after 72 h rats with marked hyperglycemia (fasting blood glucose ≥250 mg/dl) were selected and used for the study.

#### Treatment schedule

Total of 36 Wistar rats were used (30 Diabetic surviving and 06 nondiabetics). The rats were divided into six groups of 6 animals (*n* = 6) each as follows- The solution of APSAE was prepared with 1 % gum acacia, an emulsifying agent. Glibenclamide was served as a reference standard. Group-I (Nondiabetic Control) animals were received only 1 % gum acacia (1 ml/kg/day, p.o.). Group-II (Diabetic Control) animals were diabetic and received 1 % gum acacia (1 ml/kg/day, p.o.). Group-III (Diabetic + Glibenclamide) animals were diabetic and received glibenclamide (0.25 mg/kg/day, p.o.) (Sun Pharmaceuticals Ltd, India). Groups IV, V, VI animals were diabetic and received three different doses of APSAE 50, 100 and 200 mg/kg, p.o. respectively. All the animals received above treatment for 30 days.

#### Evaluation of antihyperglycaemic activity

Antihyperglycaemic activity of APSAE was evaluated by estimation of blood glucose levels and body weight measurement on 1st, 10th, 20th and 30th day of the study by the glucose oxidase/peroxidase (GOD/POD) (Trinder 1969) method using a standard kit obtained from Span Diagnostics, India.

#### Evaluation of antihyperlipidaemic activity

At the end of the experiment, the animals from each group were sacrificed by cervical dislocation and blood and organs were collected to estimate various biochemical and histological studies (Chakrabarti et al. 2005). Blood was collected from the heart and allowed to clot and the serum was separated by centrifuged at 3,500 rpm for 10 min. Serum was assayed either immediately or stored at -20°C. The tissue like pancreas was collected and used for histological studies. Serum samples were analyzed spectrophotometrically for triglycerides, total cholesterol, high density lipoprotein (HDL-C), using their respective kits UV- visible spectrophotometer (Jasco V-630, Japan), VLDL-C and LDL-C were calculated as per Friedwald’s equation (Richterich and Colmbo 1981).$$ \matrix{ {{\text{VLDL}} = {\text{TG}}/{5}}; \\ {{\text{LDL}} = {\text{TC}} - \left( {{\text{HDL}} + {\text{VLDL}}} \right)} \\ } $$.

(Alayash et al. 1988; Burstein et al. 1970; Friedwald et al. 1972).

#### Estimation of glycated hemoglobin

After 30 days of treatment, the 12 h fasted rats were sacrificed by cervical decapitation, blood was withdrawn by retro orbital puncture under light ether anesthesia and the glycated hemoglobin was estimated (Sadasivam and Manickam 1996).

### Statistical analysis

The results were expressed as mean ± S.E.M., statistical difference was doone by using one-way analysis of varience (ANOVA) followed by Dunnette’s multiple comparison test. A difference in the mean P value <0.05 was considered as statistically significant.

## Results

### Preliminary phytochemical Screening

The study showed the presence of steroids, terpenoids, alkaloids, tannins, phenolic compounds, flavonoids, Sugars and amino acids.

### Acute toxicity study

Oral administration of APSAE was found safe up to dose of 2,000 mg/kg, p.o. produced no signs of toxicity. However, from 5 g/kg APSAE caused slow movement of animal, decreased aggressiveness, altered touch and pain sensibility but did not cause any negative behavioral changes such as excitement, respiratory distress, convulsions or coma. No mortality was observed up to 14 days. Hence, the median lethal dose (LD_50_) of the APSAE was then greater than 2,000 mg/kg body weight. Therefore doses 50,100 and 200 mg/kg b.wt. were selected for all in vivo experiments.

### Effect of APSAE in normoglycaemic rats

The results from the study exhibited that there was no significant effect observed on normoglycaemic rats when treated with the single dose of APSAE at 50,100 and 200 mg/kg b.wt. (Table 1).

**Table 1:** Effect of APSAE on blood glucose level (BGL) of normoglycaemic rats

Group Treatment (*n* = 6)	Fasting plasma glucose level (mg/dl) at (hrs)				
	0	1	2	3	4
I Normal	94.00 ± 0.36	94.16 ± 0.30	91.50 ± 0.76	91.16 ± 0.47	91.66 ± 0.33
II Glibenclamide	93.83 ± 0.30	93.50 ± 0.22	90.33 ±0.71	88.83 ± 0.40*	88.00 ± 0.44**
III APSAE	93.66 ± 0.33	93.33 ± 0.21	92.00 ± 0.36	91.16 ± 0.30	90.66 ± 0.21
IV APSAE	94.33 ± 0.33	94.00 ± 0.36	91.66 ± 0.66	90.66 ± 0.49	90.16 ± 0.30*
V APSAE	94.16 ± 0.98	93.83 ± 0.40	90.50 ± 0.42	90.16 ± 0.30	89.83 ± 0.40**

*P < 0.05, **P < 0.01 Values are Mean ± SEM, *n* = 6, when compared with normal by using one way ANOVA followed by Dunnette’s multiple comparison test

### Oral glucose tolerance test in normal rats (OGTT)

The results from the study indicated that the APSAE at 50,100 and 200 mg/kg and glibenclamide (0.25 mg/kg) reduced the blood glucose level (hyperglycemia due to glucose load 2 g/kg p.o.) significantly after 3 h of oral administration, when compared to diabetic control group (Table 2).

**Table 2:** Effect of APSAE on oral glucose tolerance test in STZ-induced diabetic rats (OGTT)

Group Treatment (*n* = 6)	Fasting plasma glucose level (mg/dl) at (hrs)				
	0	1	2	3	4
I Normal	101.17 ± 1.66	126.17 ± 1.95	136.50 ± 1.05	146.00 ± 0.68	155.17 ± 0.65
II Diabetic control	258.00 ± 1.06	268.33 ± 1.25	280.17 ± 1.24	290.00 ± 0.96	299.00 ± 0.5
III Diabetic + glibenclamide	257.67 ± 1.22	267.00 ± 1.12	277.00 ± 0.96	284.17 ± 1.01*	265.67 ± 0.49**
IV Diabetic + APSAE	259.50 ± 1.05	269.33 ± 1.14	279.00 ± 0.96	289.00 ± 0.96	274.17 ± 1.16*
V Diabetic + APSAE	257.67 ± 0.84	268.00 ± 0.93	277.17 ± 1.01	287.33 ± 0.66	270.83 ± 1.49*
VI Diabetic + APSAE	260.17 ± 0.60	270.17 ± 0.60	276.67 ± 0.61	284.83 ± 0.47*	266.17 ± 0.47**

*P < 0.05, **P < 0.01 Values are Mean ± SEM, *n* = 6, when compared with diabetic control by using one way ANOVA followed by Dunnette’s multiple comparison test

### Antihyperglycaemic activity

The results from the repeated administration of APSAE daily up to 30 days exhibited significant antihyperglycaemic activity in stz-induced diabetic rats, whilst there was no significant effect observed on normoglycaemic rats. However, at the end of 30 days of treatment, there was a 74.27 %, 69.55 %, 71.45 % and 72.66 % (*p* < 0.01) decrease of serum glucose levels with the glibenclamide and APSAE (50,100 and 200 mg/kg) respectively when compared with diabetic control group (Table 3).

**Table 3:** Antihyperglycaemic effect of APSAE on blood glucose level (BGL) of STZ-induced diabetic rats

Group Treatment (*n* = 6)	Fasting plasma glucose concentration (mg/dl) at			
	1st day	10th day	20th day	30th day
I Normal	98.66 ± 3.94	101.50 ± 4.54	101.17 ± 5.30	103.17 ± 4.57
II Diabetic control	277.83 ± 1.50	372.17 ± 7.90	398.50 ± 6.28	413.33 ± 2.80
III Diabetic + glibenclamide	295.17 ± 1.37	154.67 ± 10.19**	126.67 ± 1.70**	106.33 ± 1.28**
IV Diabetic + APSAE	302.17 ± 1.74	174.33 ± 3.29*	141.67 ± 1.70*	125.83 ± 0.60*
V Diabetic + APSAE	300.83 ± 1.53	169.50 ± 4.40*	138.33 ± 1.94*	118.00 ± 0.57**
VI Diabetic + APSAE	302.50 ± 4.54	163.83 ± 3.68*	127.33 ± 1.58**	113.00 ± 1.03**

*P < 0.05, **P < 0.01 Values are Mean ± SEM, *n* = 6, when compared with diabetic control by using one way ANOVA followed by Dunnette’s multiple comparison test

### Antihyperlipidaemic activity

The results from the repeated administration of APSAE daily up to 30 days exhibited significant reduction in lipid profile in stz-induced diabetic rats. Lipid profile of animals showed significant reductions (*p* < 0.01) of 13.82 %, 18.08 % and 22.34 % CHL (cholesterol), 44.21 %, 51.57 % and 60.00 % LDL, 11.60 %, 18.13 % and 18.86 % VLDL (Very Low density lipoproteins) and 27.43 %, 30.08 % and 31.85 % TG after treatment with APSAE 50,100 and 200 mg/kg respectively when compared with diabetic control rats. There was also a significant (*p* < 0.01) increase of 54.12 %, 66.62 % and 70.75 % HDL in the APSAE treated diabetic rats in comparison of diabetic control rats, where a fall in HDL level (Table 4).

**Table 4:** Effect of APSAE on serum lipid profile in STZ-induced diabetic rats

Group Treatment (*n* = 6)	Body Cholesterol	LDL	HDL	VLDL	Triglycerides
	in (mg/dl)				
I Normal	66.83 ± 0.47	23.33 ± 0.42	11.50 ± 0.42	16.66 ± 0.30	66.00 ± 0.57
II Diabetic control	94.00 ± 0.73	95.50 ± 0.76	8.00 ± 0.51	23.00 ± 0.36	113.17 ± 0.60
III Diabetic + glibenclamide	69.66 ± 0.49**	36.83 ± 0.60**	12.16 ± 0.30**	17.83 ± 0.65**	75.50 ± 0.76**
IV Diabetic + APSAE	81.16 ± 0.47*	53.16 ± 0.47**	12.33 ± 0.33**	20.33 ± 0.42*	82.50 ± 0.76**
V Diabetic + APSAE	77.00 ± 0.57**	46.66 ± 0.49**	13.33 ± 0.33**	18.83 ± 0.40**	79.50 ± 0.78**
VI Diabetic + APSAE	73.50 ± 0.42**	38.83 ± 0.60**	13.66 ± 0.42**	18.66 ± 0.21**	77.50 ± 0.38**

*P < 0.05, **P < 0.01 Values are Mean ± SEM, *n* = 6, when compared with diabetic control by using one way ANOVA followed by Dunnette’s multiple comparison test

### Changes in body weight

At the end of 30 days treatment, the body weight of normal rats, APSAE and standard drug treated group, increased significantly, whereas body weight of diabetic control group decreased (Table 5).

**Table 5:** Effect of APSAE on body weight in STZ-induced diabetic rats

Group Treatment (*n* = 6)	Changes in body weight (gm) at			
	1st day	10th day	20th day	30th day
I Normal	160.00 ± 0.83	167.00 ± 0.85	176.50 ± 0.76	185.50 ± 0.76
II Diabetic control	161.17 ± 0.94	155.33 ± 1.30	149.83 ± 1.13	140.17 ± 1.77
III Diabetic + glibenclamide	159.50 ± 1.89	163.50 ± 1.23*	170.50 ± 0.61**	179.17 ± 0.70**
IV Diabetic + APSAE	159.83 ± 2.07	163.67 ± 1.87*	169.67 ± 0.98**	176.50 ± 0.84**
V Diabetic + APSAE	158.67 ± 5.04	165.17 ± 1.62*	171.73 ± 1.25**	177.83 ± 0.79**
VI Diabetic + APSAE	159.17 ± 1.13	166.67 ± 0.84*	173.17 ± 1.13**	180.33 ± 0.76**

*P < 0.05, **P < 0.01 Values are Mean ± SEM, *n* = 6, when compared with diabetic control by using one way ANOVA followed by Dunnette’s multiple comparison test

### Changes of serum glycosylated hemoglobin

After 30 days of treatment with APSAE, it was observed that animals treated with APSAE showed a significant decrease in glycosylated hemoglobin levels when compared to diabetic control groups (Table 6).

**Table 6:** Effect of APSAE on glycosylated Hb and change in body weights of normal and diabetic rats

Group Treatment (*n* = 6)	HbA1c mg/gm Hb
I Normal	6.45 ± 0.17
II Diabetic control	10.83 ± 0.11
III Diabetic + glibenclamide	7.50 ± 0.005**
IV Diabetic + APSAE	8.40 ± 0.38**
V Diabetic + APSAE	8.16 ± 0.04**
VI Diabetic + APSAE	7.80 ± 0.07**

*P < 0.05, **P < 0.01 Values are Mean ± SEM, *n* = 6, when compared with diabetic control by using one way ANOVA followed by Dunnette’s multiple comparison test

## Discussion

The use of traditional medicine and medicinal plants in most developing countries, as a normative basis for the maintenance of good health has been widely observed (Tiwari and Madhusudanarao 2002). Diabetes mellitus is probably the fastest growing metabolic disease in the world and as knowledge of the heterogeneous nature of the disease increases so does the need for more challenging and appropriate therapies. Traditional plant remedies have been used for centuries in the treatment of diabetes (Akhtar and Ali 1984; Kesari et al. 2005; 2007; Rai et al. 2007), but only a few have been scientifically evaluated. Therefore, we have investigated the effect of *Adenanthera pavonina* seed aqueous extract on glycemic control and serum lipid profile in STZ-induced diabetic rats. APSAE showed a dose dependent effect on fasting blood glucose at 50,100 and 200 mg/kg b.wt. in diabetic rats. So, detailed studies were carried out with the dose of 50,100 and 200 mg APSAE mg/kg b.wt. The capacity of APSAE to decrease the elevated blood glucose to normal level is an essential trigger for the liver to revert to its normal homeostasis during experimental diabetes. Lower levels of total hemoglobin observed in diabetic rats might be due to the increased formation of HbA1c. In uncontrolled or poorly controlled diabetes, there is an increased glycosylation of a number of proteins including hemoglobin and crystalline of lens (Alberti and Keen 1982). HbA1c was found to increase in patients with diabetes mellitus and the amount of increase was directly proportional to the fasting blood glucose levels (Pari and Saravanan 2002) therefore, measurement of HbA1c is supposed to be very sensitive index for glycemic control. Treatment with APSAE showed a significant decrease in the glycated hemoglobin levels, which could be due to an improvement in insulin secretion. Induction of diabetes with STZ is associated with the characteristic loss of body weight, which is due to increased muscle wasting (Swanston-Flatt et al. 1990), and due to loss of tissue proteins (Chatterjea and Shinde 1976). Diabetic rats treated with the APSAE showed an increase in body weight when compared to the untreated diabetic rats which may be due to its protective effect in controlling muscle wasting i.e. reversal of gluconeogenesis and may also be due to the improvement in glycemic control. Increased levels of triglycerides and cholesterol during diabetes lead to cardiovascular complications. In this study, STZ-induced diabetic mellitus characterized by hyperglycemia caused a significant rise in serum lipids. These findings indicate that diabetes mellitus is accompanied by increased risk of atherosclerosis and coronary artery diseases. Lowering of serum lipid levels through dietary or drug therapy seems to be associated with a decrease in the risk of vascular disease (Rhoads et al. 1976). In the present study, the APSAE significantly reduced the triglyceride, total cholesterol, LDL and VLDL cholesterol levels with an increase of HDL cholesterol in treated diabetic rats as compared to untreated diabetic rats so, these changes are could be beneficial in preventing diabetic complications as well as in improving lipid metabolism in diabetics (Gupta et al. 2005). The significant control of the levels of serum lipids in the APSAE treated diabetic rats could be directly attributed to improvement in glycemic control upon APSAE therapy. Hence, these findings demonstrate that *Adenanthera pavonina* could be beneficial to treat diabetes mellitus and complications owing to its antihyperglycaemic and lipid lowering effect. Further studies are necessary to substantiate above claim and to work out exact mechanism of action involved in antihyperglycaemic and antihyperlipidaemic activity of this plant.
